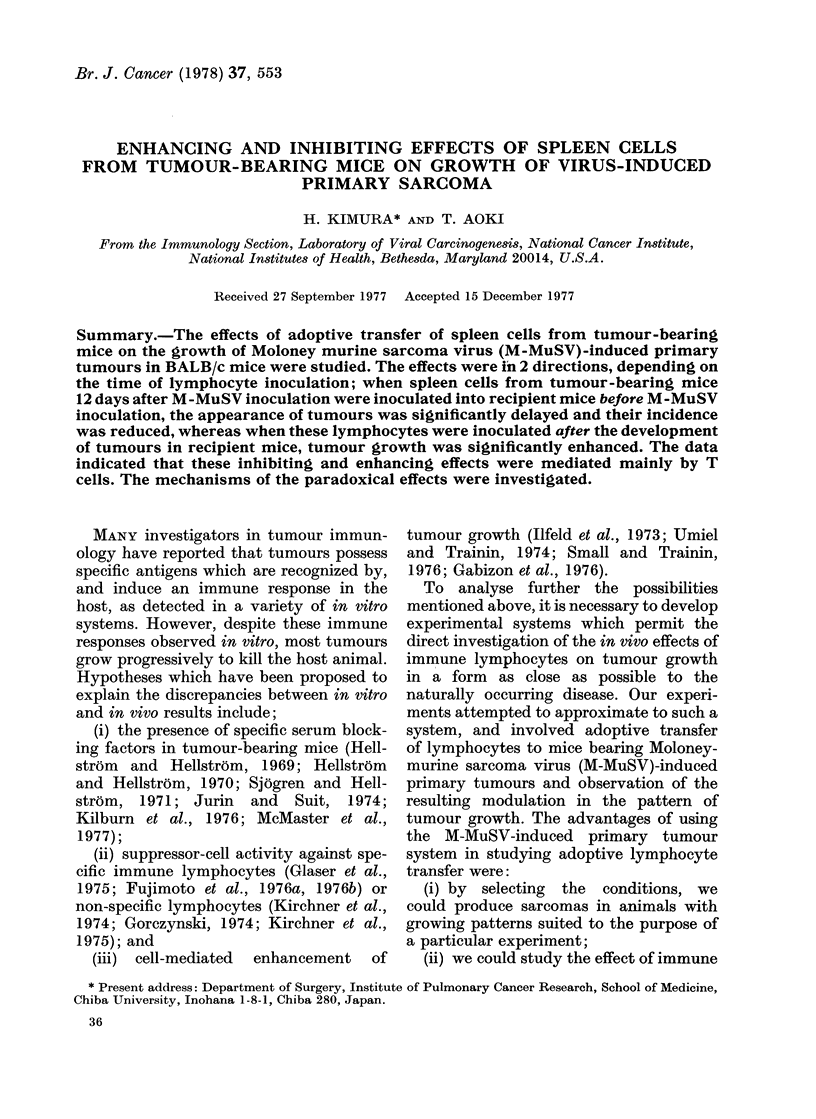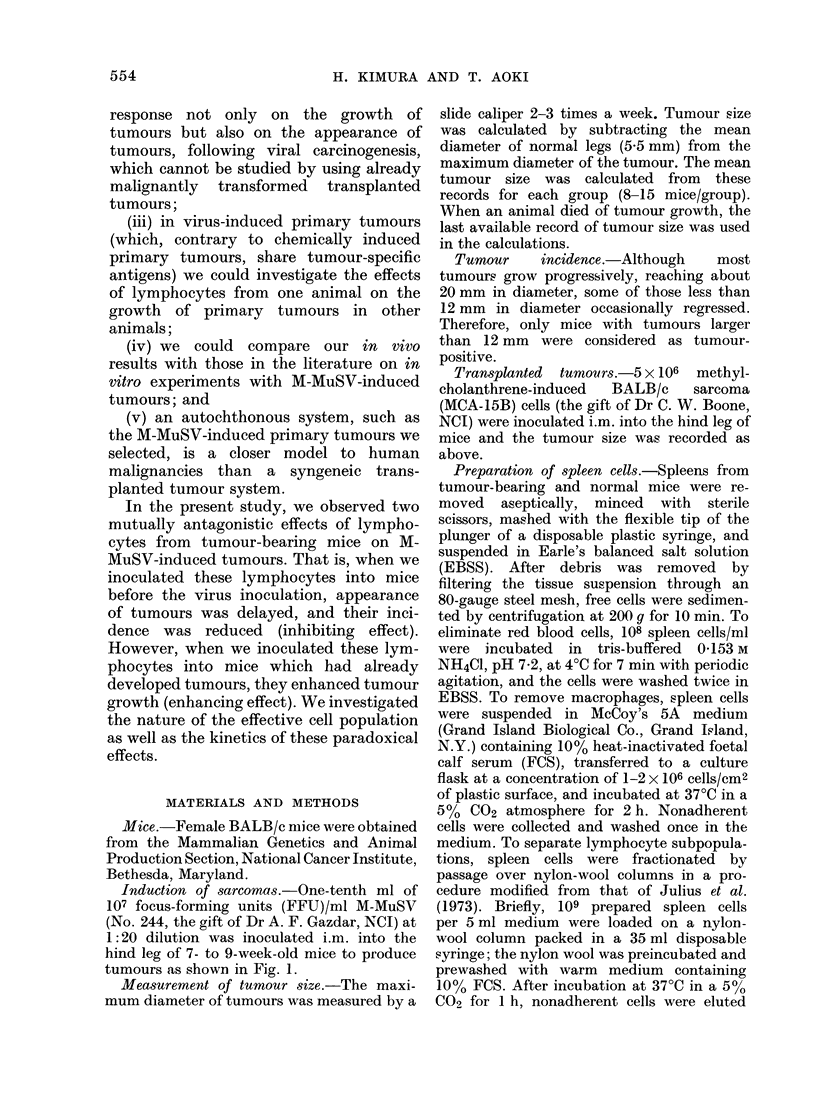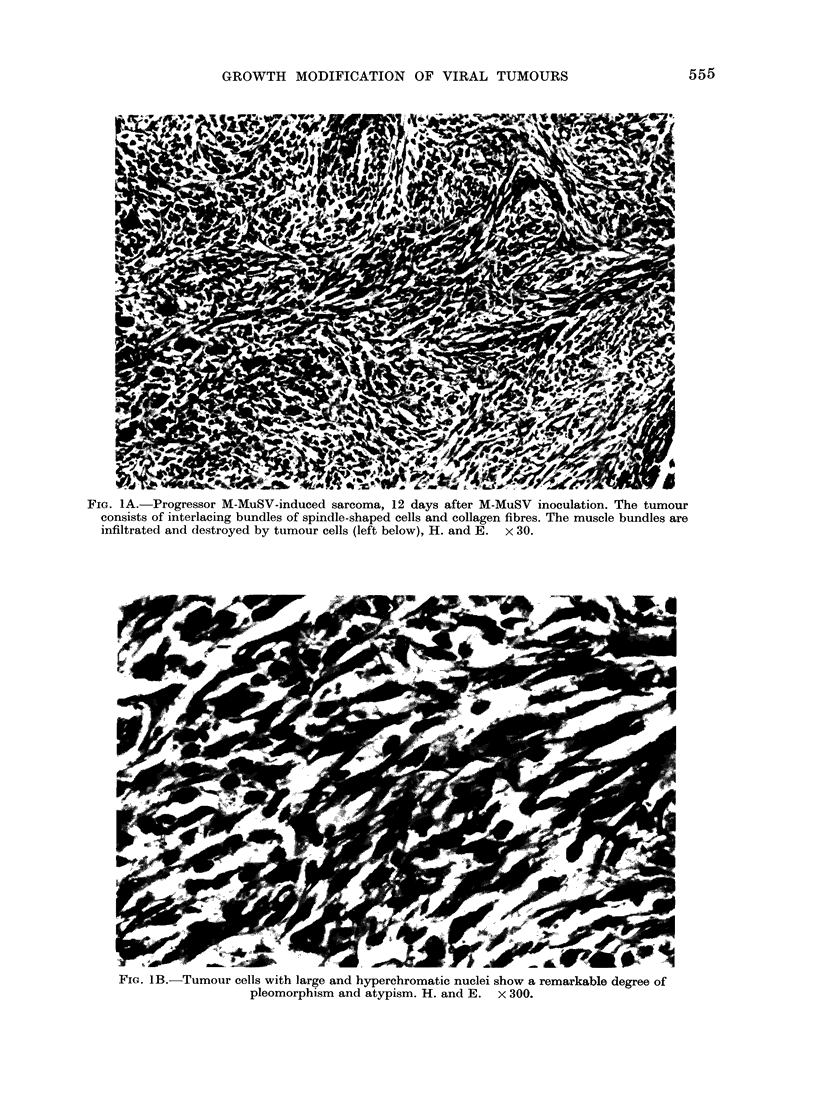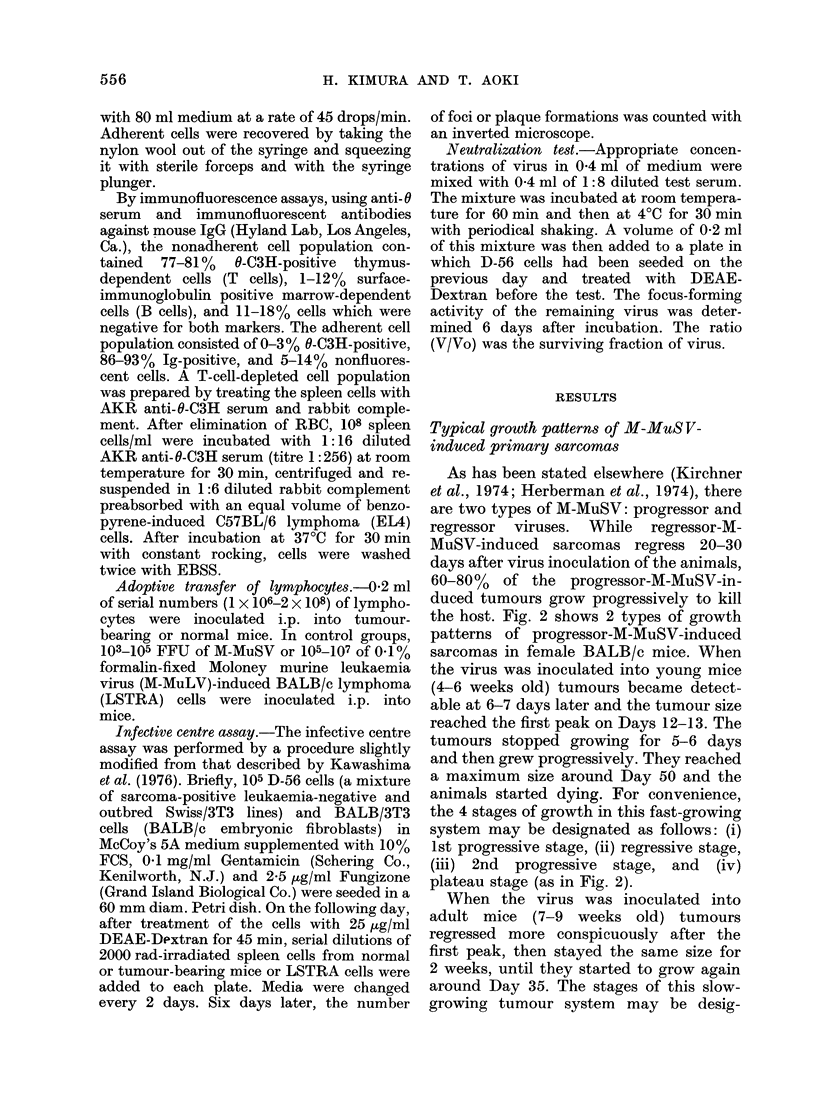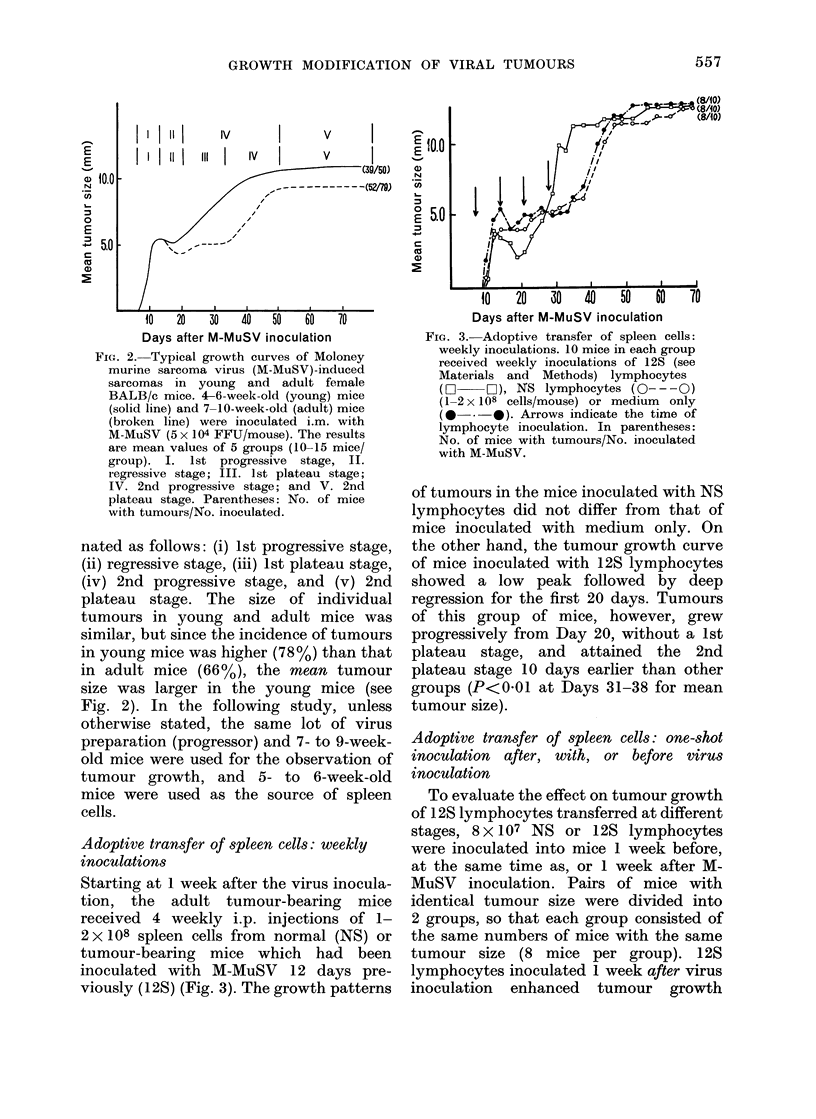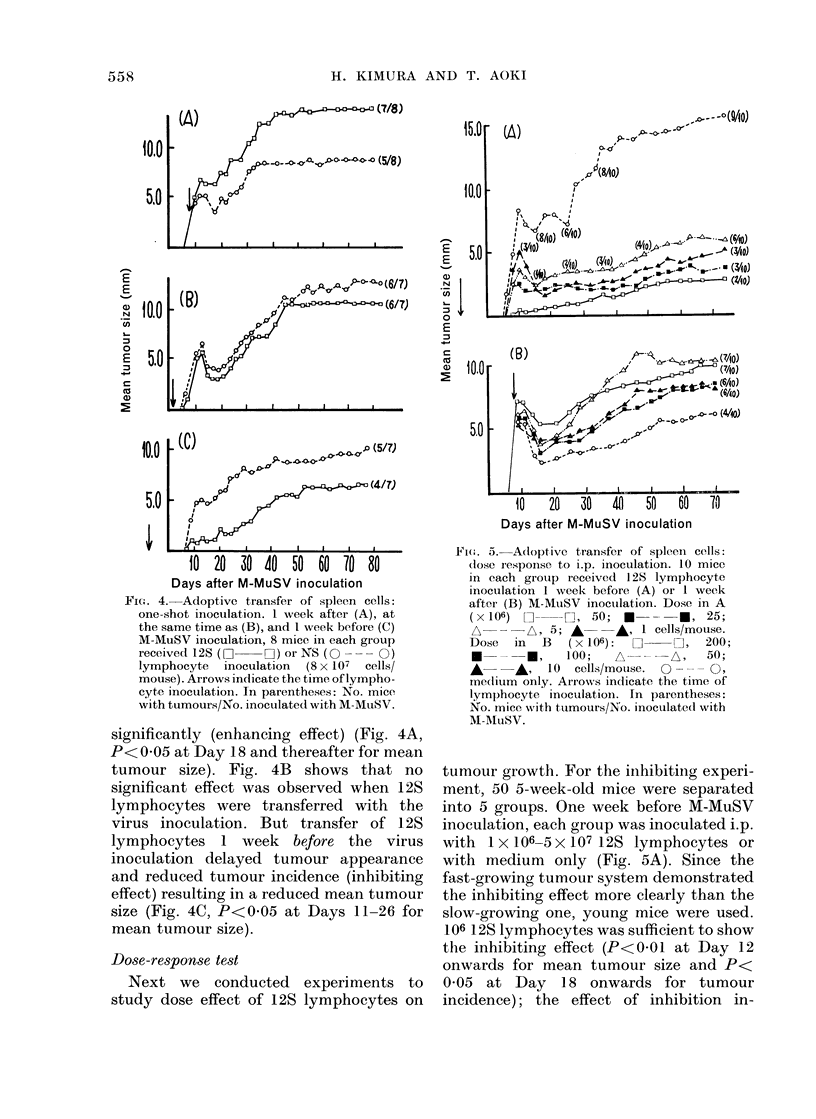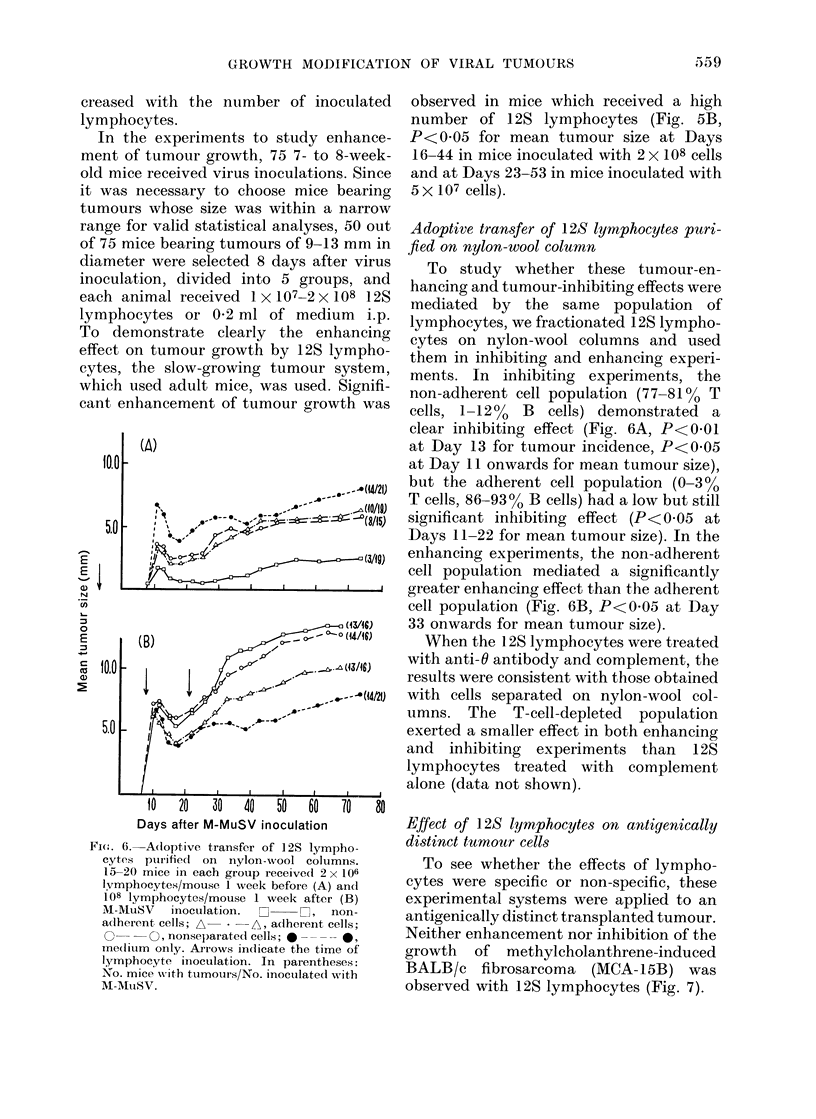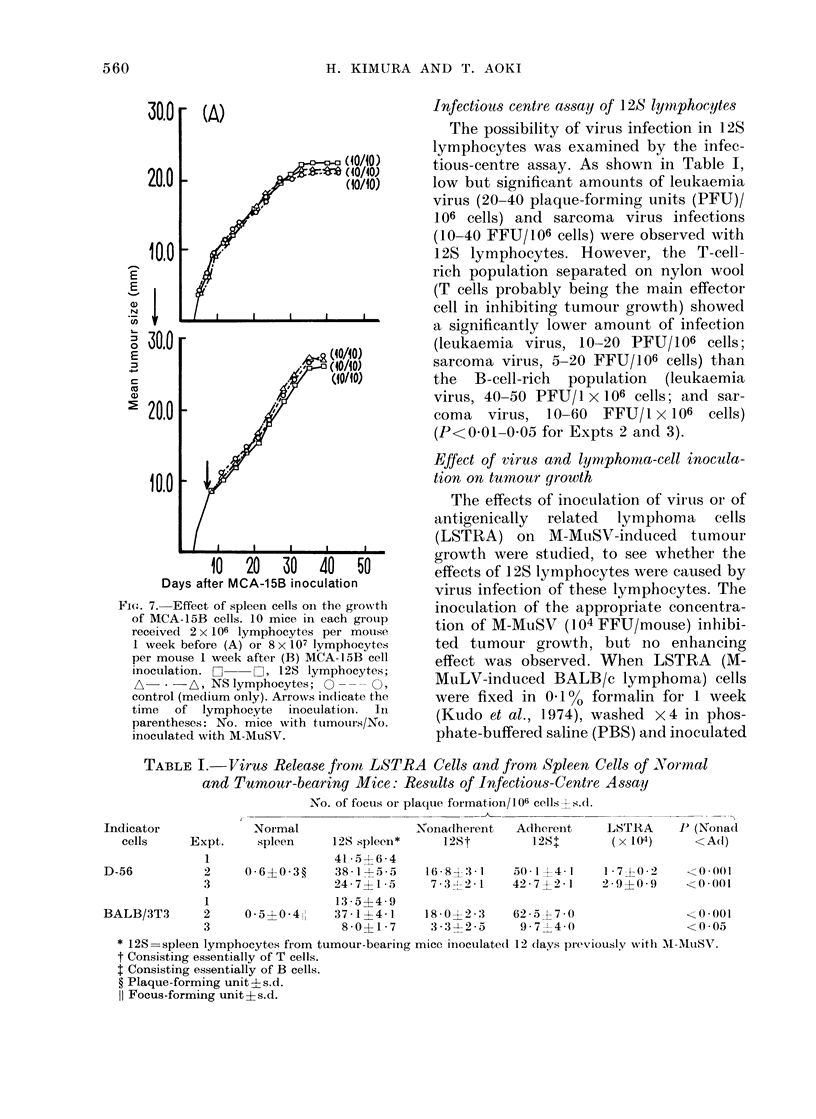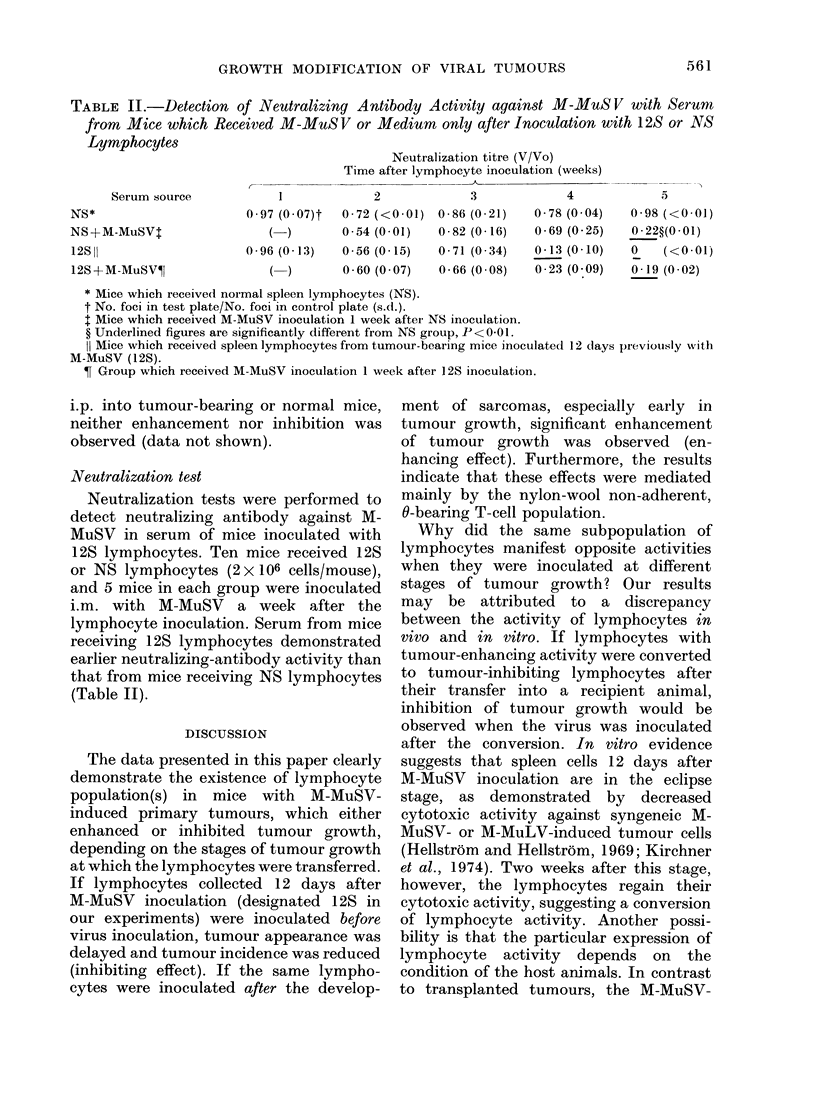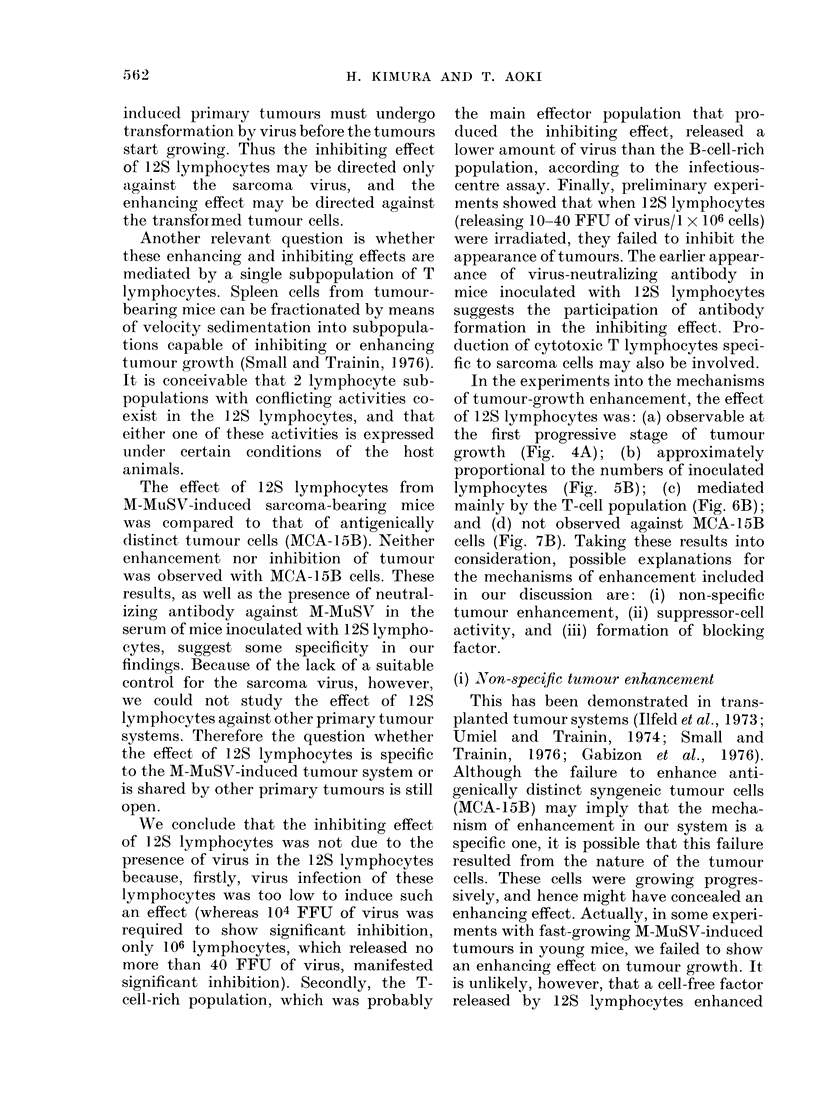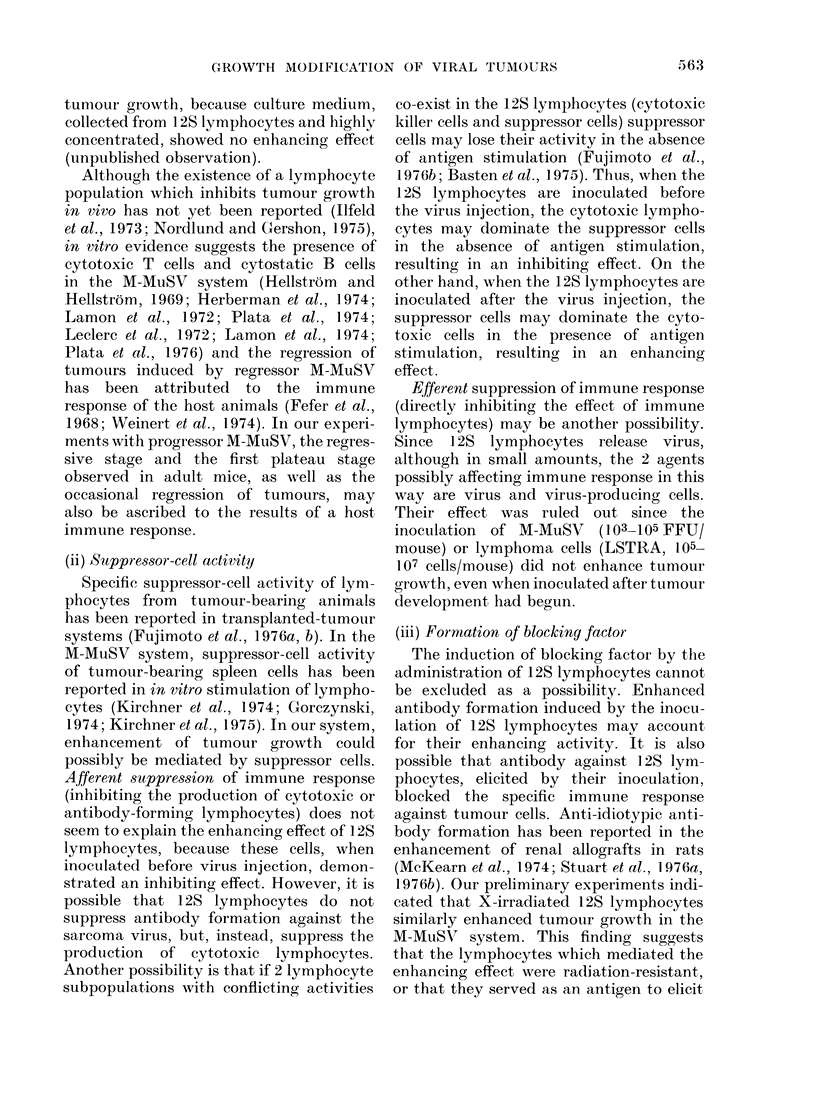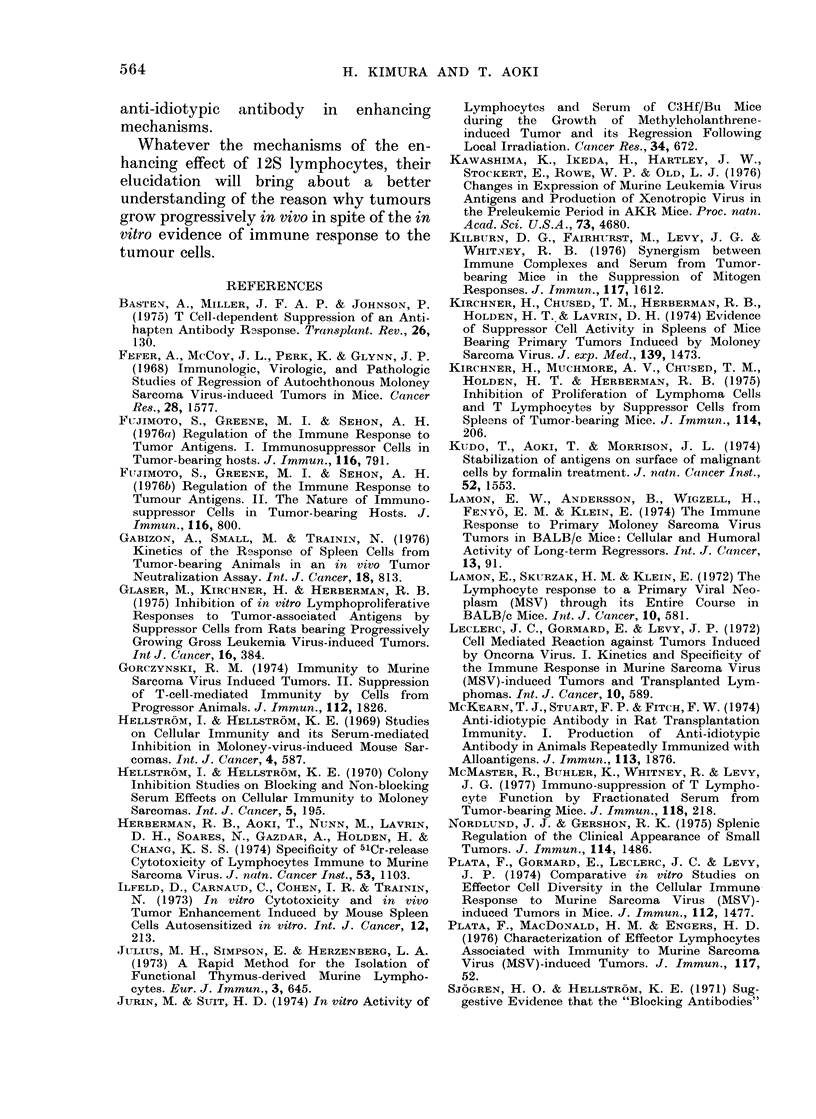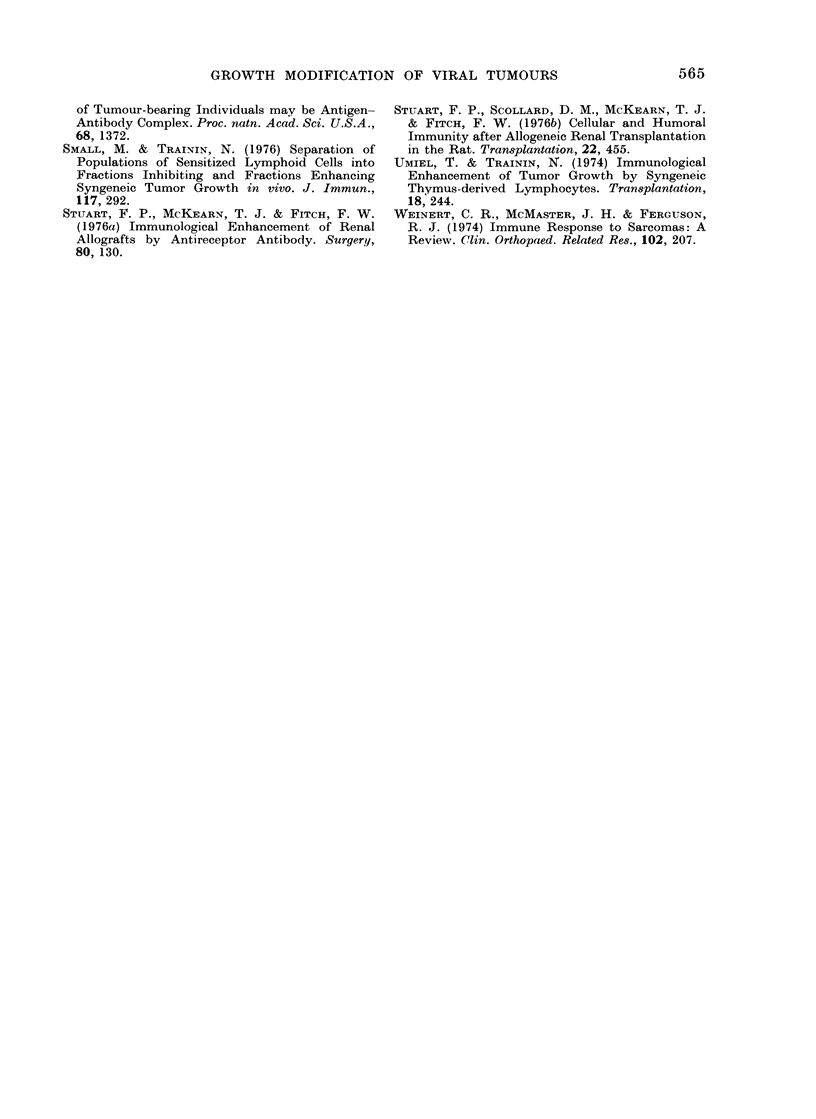# Enhancing and inhibiting effects of spleen cells from tumour-bearing mice on growth of virus-induced primary sarcoma.

**DOI:** 10.1038/bjc.1978.84

**Published:** 1978-04

**Authors:** H. Kimura, T. Aoki

## Abstract

**Images:**


*Br. J. Cancer* (1978) **37**, 553

### ENHANCING AND INHIBITING EFFECTS OF SPLEEN CELLS FROM TUMOUR-BEARING MICE ON GROWTH OF VIRUS-INDUCED PRIMARY SARCOMA

H. KIMURA* AND T. AOKI

*From the Immunology Section, Laboratory of Viral Carcinogenesis, National Cancer Institute, National Institutes of Health, Bethesda, Maryland* 20014, *U.S.A.*

Received 27 September 1977 Accepted 15 December 1977

**Summary.—The effects of adoptive transfer of spleen cells from tumour-bearing mice on the growth of Moloney murine sarcoma virus (M-MuSV)-induced primary tumours in BALB/c mice were studied. The effects were in 2 directions, depending on the time of lymphocyte inoculation; when spleen cells from tumour-bearing mice 12 days after M-MuSV inoculation were inoculated into recipient mice *before* M-MuSV inoculation, the appearance of tumours was significantly delayed and their incidence was reduced, whereas when these lymphocytes were inoculated *after* the development of tumours in recipient mice, tumour growth was significantly enhanced. The data indicated that these inhibiting and enhancing effects were mediated mainly by T cells. The mechanisms of the paradoxical effects were investigated.**

MANY investigators in tumour immunology have reported that tumours possess specific antigens which are recognized by, and induce an immune response in the host, as detected in a variety of *in vitro* systems. However, despite these immune responses observed *in vitro*, most tumours grow progressively to kill the host animal. Hypotheses which have been proposed to explain the discrepancies between *in vitro* and *in vivo* results include;

(i) the presence of specific serum blocking factors in tumour-bearing mice (Hellström and Hellström, 1969; Hellström and Hellström, 1970; Sjögren and Hellström, 1971; Jurin and Suit, 1974; Kilburn *et al.*, 1976; McMaster *et al.*, 1977);

(ii) suppressor-cell activity against specific immune lymphocytes (Glaser *et al.*, 1975; Fujimoto *et al.*, 1976*a*, 1976*b*) or non-specific lymphocytes (Kirchner *et al.*, 1974; Gorczynski, 1974; Kirchner *et al.*, 1975); and

(iii) cell-mediated enhancement of tumour growth (Ilfeld *et al.*, 1973; Umiel and Trainin, 1974; Small and Trainin, 1976; Gabizon *et al.*, 1976).

To analyse further the possibilities mentioned above, it is necessary to develop experimental systems which permit the direct investigation of the *in vivo* effects of immune lymphocytes on tumour growth in a form as close as possible to the naturally occurring disease. Our experiments attempted to approximate to such a system, and involved adoptive transfer of lymphocytes to mice bearing Moloney-murine sarcoma virus (M-MuSV)-induced primary tumours and observation of the resulting modulation in the pattern of tumour growth. The advantages of using the M-MuSV-induced primary tumour system in studying adoptive lymphocyte transfer were:

(i) by selecting the conditions, we could produce sarcomas in animals with growing patterns suited to the purpose of a particular experiment;

(ii) we could study the effect of immune

* Present address: Department of Surgery, Institute of Pulmonary Cancer Research, School of Medicine, Chiba University, Inohana 1-8-1, Chiba 280, Japan.

response not only on the growth of tumours but also on the appearance of tumours, following viral carcinogenesis, which cannot be studied by using already malignantly transformed transplanted tumours;

(iii) in virus-induced primary tumours (which, contrary to chemically induced primary tumours, share tumour-specific antigens) we could investigate the effects of lymphocytes from one animal on the growth of primary tumours in other animals;

(iv) we could compare our *in vivo* results with those in the literature on *in vitro* experiments with M-MuSV-induced tumours; and

(v) an autochthonous system, such as the M-MuSV-induced primary tumours we selected, is a closer model to human malignancies than a syngeneic transplanted tumour system.

In the present study, we observed two mutually antagonistic effects of lymphocytes from tumour-bearing mice on M-MuSV-induced tumours. That is, when we inoculated these lymphocytes into mice before the virus inoculation, appearance of tumours was delayed, and their incidence was reduced (inhibiting effect). However, when we inoculated these lymphocytes into mice which had already developed tumours, they enhanced tumour growth (enhancing effect). We investigated the nature of the effective cell population as well as the kinetics of these paradoxical effects.

### MATERIALS AND METHODS

*Mice.*—Female BALB/c mice were obtained from the Mammalian Genetics and Animal Production Section, National Cancer Institute, Bethesda, Maryland.

*Induction of sarcomas.*—One-tenth ml of $10^7$ focus-forming units (FFU)/ml M-MuSV (No. 244, the gift of Dr A. F. Gazdar, NCI) at 1:20 dilution was inoculated i.m. into the hind leg of 7- to 9-week-old mice to produce tumours as shown in Fig. 1.

*Measurement of tumour size.*—The maximum diameter of tumours was measured by a slide caliper 2–3 times a week. Tumour size was calculated by subtracting the mean diameter of normal legs (5·5 mm) from the maximum diameter of the tumour. The mean tumour size was calculated from these records for each group (8–15 mice/group). When an animal died of tumour growth, the last available record of tumour size was used in the calculations.

*Tumour incidence.*—Although most tumours grow progressively, reaching about 20 mm in diameter, some of those less than 12 mm in diameter occasionally regressed. Therefore, only mice with tumours larger than 12 mm were considered as tumour-positive.

*Transplanted tumours.*—$5 \times 10^6$ methylcholanthrene-induced BALB/c sarcoma (MCA-15B) cells (the gift of Dr C. W. Boone, NCI) were inoculated i.m. into the hind leg of mice and the tumour size was recorded as above.

*Preparation of spleen cells.*—Spleens from tumour-bearing and normal mice were removed aseptically, minced with sterile scissors, mashed with the flexible tip of the plunger of a disposable plastic syringe, and suspended in Earle's balanced salt solution (EBSS). After debris was removed by filtering the tissue suspension through an 80-gauge steel mesh, free cells were sedimented by centrifugation at 200 *g* for 10 min. To eliminate red blood cells, $10^8$ spleen cells/ml were incubated in tris-buffered 0·153 M $NH_4Cl$, pH 7·2, at 4°C for 7 min with periodic agitation, and the cells were washed twice in EBSS. To remove macrophages, spleen cells were suspended in McCoy's 5A medium (Grand Island Biological Co., Grand Island, N.Y.) containing 10% heat-inactivated foetal calf serum (FCS), transferred to a culture flask at a concentration of $1–2 \times 10^6$ cells/cm$^2$ of plastic surface, and incubated at 37°C in a 5% $CO_2$ atmosphere for 2 h. Nonadherent cells were collected and washed once in the medium. To separate lymphocyte subpopulations, spleen cells were fractionated by passage over nylon-wool columns in a procedure modified from that of Julius *et al.* (1973). Briefly, $10^9$ prepared spleen cells per 5 ml medium were loaded on a nylon-wool column packed in a 35 ml disposable syringe; the nylon wool was preincubated and prewashed with warm medium containing 10% FCS. After incubation at 37°C in a 5% $CO_2$ for 1 h, nonadherent cells were eluted

FIG. 1A.—Progressor M-MuSV-induced sarcoma, 12 days after M-MuSV inoculation. The tumour consists of interlacing bundles of spindle-shaped cells and collagen fibres. The muscle bundles are infiltrated and destroyed by tumour cells (left below), H. and E. ×30.

FIG. 1B.—Tumour cells with large and hyperchromatic nuclei show a remarkable degree of pleomorphism and atypism. H. and E. ×300.

with 80 ml medium at a rate of 45 drops/min. Adherent cells were recovered by taking the nylon wool out of the syringe and squeezing it with sterile forceps and with the syringe plunger.

By immunofluorescence assays, using anti-$\theta$ serum and immunofluorescent antibodies against mouse IgG (Hyland Lab, Los Angeles, Ca.), the nonadherent cell population contained 77–81% $\theta$-C3H-positive thymus-dependent cells (T cells), 1–12% surface-immunoglobulin positive marrow-dependent cells (B cells), and 11–18% cells which were negative for both markers. The adherent cell population consisted of 0–3% $\theta$-C3H-positive, 86–93% Ig-positive, and 5–14% nonfluorescent cells. A T-cell-depleted cell population was prepared by treating the spleen cells with AKR anti-$\theta$-C3H serum and rabbit complement. After elimination of RBC, $10^8$ spleen cells/ml were incubated with 1:16 diluted AKR anti-$\theta$-C3H serum (titre 1:256) at room temperature for 30 min, centrifuged and resuspended in 1:6 diluted rabbit complement preabsorbed with an equal volume of benzopyrene-induced C57BL/6 lymphoma (EL4) cells. After incubation at 37°C for 30 min with constant rocking, cells were washed twice with EBSS.

*Adoptive transfer of lymphocytes.*—0·2 ml of serial numbers ($1 \times 10^6$–$2 \times 10^8$) of lymphocytes were inoculated i.p. into tumour-bearing or normal mice. In control groups, $10^3$–$10^5$ FFU of M-MuSV or $10^5$–$10^7$ of 0·1% formalin-fixed Moloney murine leukaemia virus (M-MuLV)-induced BALB/c lymphoma (LSTRA) cells were inoculated i.p. into mice.

*Infective centre assay.*—The infective centre assay was performed by a procedure slightly modified from that described by Kawashima *et al.* (1976). Briefly, $10^5$ D-56 cells (a mixture of sarcoma-positive leukaemia-negative and outbred Swiss/3T3 lines) and BALB/3T3 cells (BALB/c embryonic fibroblasts) in McCoy's 5A medium supplemented with 10% FCS, 0·1 mg/ml Gentamicin (Schering Co., Kenilworth, N.J.) and 2·5 $\mu$g/ml Fungizone (Grand Island Biological Co.) were seeded in a 60 mm diam. Petri dish. On the following day, after treatment of the cells with 25 $\mu$g/ml DEAE-Dextran for 45 min, serial dilutions of 2000 rad-irradiated spleen cells from normal or tumour-bearing mice or LSTRA cells were added to each plate. Media were changed every 2 days. Six days later, the number of foci or plaque formations was counted with an inverted microscope.

*Neutralization test.*—Appropriate concentrations of virus in 0·4 ml of medium were mixed with 0·4 ml of 1:8 diluted test serum. The mixture was incubated at room temperature for 60 min and then at 4°C for 30 min with periodical shaking. A volume of 0·2 ml of this mixture was then added to a plate in which D-56 cells had been seeded on the previous day and treated with DEAE-Dextran before the test. The focus-forming activity of the remaining virus was determined 6 days after incubation. The ratio (V/Vo) was the surviving fraction of virus.

### RESULTS

### *Typical growth patterns of M-MuSV-induced primary sarcomas*

As has been stated elsewhere (Kirchner *et al.*, 1974; Herberman *et al.*, 1974), there are two types of M-MuSV: progressor and regressor viruses. While regressor-M-MuSV-induced sarcomas regress 20–30 days after virus inoculation of the animals, 60–80% of the progressor-M-MuSV-induced tumours grow progressively to kill the host. Fig. 2 shows 2 types of growth patterns of progressor-M-MuSV-induced sarcomas in female BALB/c mice. When the virus was inoculated into young mice (4–6 weeks old) tumours became detectable at 6–7 days later and the tumour size reached the first peak on Days 12–13. The tumours stopped growing for 5–6 days and then grew progressively. They reached a maximum size around Day 50 and the animals started dying. For convenience, the 4 stages of growth in this fast-growing system may be designated as follows: (i) 1st progressive stage, (ii) regressive stage, (iii) 2nd progressive stage, and (iv) plateau stage (as in Fig. 2).

When the virus was inoculated into adult mice (7–9 weeks old) tumours regressed more conspicuously after the first peak, then stayed the same size for 2 weeks, until they started to grow again around Day 35. The stages of this slow-growing tumour system may be desig-

FIG. 2.—Typical growth curves of Moloney murine sarcoma virus (M-MuSV)-induced sarcomas in young and adult female BALB/c mice. 4–6-week-old (young) mice (solid line) and 7–10-week-old (adult) mice (broken line) were inoculated i.m. with M-MuSV ($5 \times 10^4$ FFU/mouse). The results are mean values of 5 groups (10–15 mice/group). I. 1st progressive stage, II. regressive stage; III. 1st plateau stage; IV. 2nd progressive stage; and V. 2nd plateau stage. Parentheses: No. of mice with tumours/No. inoculated.

FIG. 3.—Adoptive transfer of spleen cells: weekly inoculations. 10 mice in each group received weekly inoculations of 12S (see Materials and Methods) lymphocytes (□——□), NS lymphocytes (○- - -○) ($1–2 \times 10^8$ cells/mouse) or medium only (●—·—●). Arrows indicate the time of lymphocyte inoculation. In parentheses: No. of mice with tumours/No. inoculated with M-MuSV.

nated as follows: (i) 1st progressive stage, (ii) regressive stage, (iii) 1st plateau stage, (iv) 2nd progressive stage, and (v) 2nd plateau stage. The size of individual tumours in young and adult mice was similar, but since the incidence of tumours in young mice was higher (78%) than that in adult mice (66%), the *mean* tumour size was larger in the young mice (see Fig. 2). In the following study, unless otherwise stated, the same lot of virus preparation (progressor) and 7- to 9-week-old mice were used for the observation of tumour growth, and 5- to 6-week-old mice were used as the source of spleen cells.

*Adoptive transfer of spleen cells: weekly inoculations*

Starting at 1 week after the virus inoculation, the adult tumour-bearing mice received 4 weekly i.p. injections of $1–2 \times 10^8$ spleen cells from normal (NS) or tumour-bearing mice which had been inoculated with M-MuSV 12 days previously (12S) (Fig. 3). The growth patterns of tumours in the mice inoculated with NS lymphocytes did not differ from that of mice inoculated with medium only. On the other hand, the tumour growth curve of mice inoculated with 12S lymphocytes showed a low peak followed by deep regression for the first 20 days. Tumours of this group of mice, however, grew progressively from Day 20, without a 1st plateau stage, and attained the 2nd plateau stage 10 days earlier than other groups ($P < 0{\cdot}01$ at Days 31–38 for mean tumour size).

*Adoptive transfer of spleen cells: one-shot inoculation after, with, or before virus inoculation*

To evaluate the effect on tumour growth of 12S lymphocytes transferred at different stages, $8 \times 10^7$ NS or 12S lymphocytes were inoculated into mice 1 week before, at the same time as, or 1 week after M-MuSV inoculation. Pairs of mice with identical tumour size were divided into 2 groups, so that each group consisted of the same numbers of mice with the same tumour size (8 mice per group). 12S lymphocytes inoculated 1 week *after* virus inoculation enhanced tumour growth

FIG. 4.—Adoptive transfer of spleen cells: one-shot inoculation. 1 week after (A), at the same time as (B), and 1 week before (C) M-MuSV inoculation, 8 mice in each group received 12S (□——□) or NS (○ - - - ○) lymphocyte inoculation ($8 \times 10^7$ cells/mouse). Arrows indicate the time of lymphocyte inoculation. In parentheses: No. mice with tumours/No. inoculated with M-MuSV.

FIG. 5.—Adoptive transfer of spleen cells: dose response to i.p. inoculation. 10 mice in each group received 12S lymphocyte inoculation 1 week before (A) or 1 week after (B) M-MuSV inoculation. Dose in A ($\times 10^6$) □——□, 50; ■- - - -■, 25; △- - - -△, 5; ▲——▲, 1 cells/mouse. Dose in B ($\times 10^6$): □——□, 200; ■- - - -■, 100; △- - - -△, 50; ▲——▲, 10 cells/mouse. ○ - - - ○, medium only. Arrows indicate the time of lymphocyte inoculation. In parentheses: No. mice with tumours/No. inoculated with M-MuSV.

significantly (enhancing effect) (Fig. 4A, $P<0{\cdot}05$ at Day 18 and thereafter for mean tumour size). Fig. 4B shows that no significant effect was observed when 12S lymphocytes were transferred with the virus inoculation. But transfer of 12S lymphocytes 1 week *before* the virus inoculation delayed tumour appearance and reduced tumour incidence (inhibiting effect) resulting in a reduced mean tumour size (Fig. 4C, $P<0{\cdot}05$ at Days 11–26 for mean tumour size).

*Dose-response test*

Next we conducted experiments to study dose effect of 12S lymphocytes on tumour growth. For the inhibiting experiment, 50 5-week-old mice were separated into 5 groups. One week before M-MuSV inoculation, each group was inoculated i.p. with $1\times10^6$–$5\times10^7$ 12S lymphocytes or with medium only (Fig. 5A). Since the fast-growing tumour system demonstrated the inhibiting effect more clearly than the slow-growing one, young mice were used. $10^6$ 12S lymphocytes was sufficient to show the inhibiting effect ($P<0{\cdot}01$ at Day 12 onwards for mean tumour size and $P<0{\cdot}05$ at Day 18 onwards for tumour incidence); the effect of inhibition increased with the number of inoculated lymphocytes.

In the experiments to study enhancement of tumour growth, 75 7- to 8-week-old mice received virus inoculations. Since it was necessary to choose mice bearing tumours whose size was within a narrow range for valid statistical analyses, 50 out of 75 mice bearing tumours of 9–13 mm in diameter were selected 8 days after virus inoculation, divided into 5 groups, and each animal received $1 \times 10^7$–$2 \times 10^8$ 12S lymphocytes or 0·2 ml of medium i.p. To demonstrate clearly the enhancing effect on tumour growth by 12S lymphocytes, the slow-growing tumour system, which used adult mice, was used. Significant enhancement of tumour growth was observed in mice which received a high number of 12S lymphocytes (Fig. 5B, $P<0{\cdot}05$ for mean tumour size at Days 16–44 in mice inoculated with $2 \times 10^8$ cells and at Days 23–53 in mice inoculated with $5 \times 10^7$ cells).

FIG. 6.—Adoptive transfer of 12S lymphocytes purified on nylon-wool columns. 15–20 mice in each group received $2 \times 10^6$ lymphocytes/mouse 1 week before (A) and $10^8$ lymphocytes/mouse 1 week after (B) M-MuSV inoculation. □——□, non-adherent cells; △— · —△, adherent cells; ○— —○, nonseparated cells; ●– – – –●, medium only. Arrows indicate the time of lymphocyte inoculation. In parentheses: No. mice with tumours/No. inoculated with M-MuSV.

*Adoptive transfer of 12S lymphocytes purified on nylon-wool column*

To study whether these tumour-enhancing and tumour-inhibiting effects were mediated by the same population of lymphocytes, we fractionated 12S lymphocytes on nylon-wool columns and used them in inhibiting and enhancing experiments. In inhibiting experiments, the non-adherent cell population (77–81% T cells, 1–12% B cells) demonstrated a clear inhibiting effect (Fig. 6A, $P<0{\cdot}01$ at Day 13 for tumour incidence, $P<0{\cdot}05$ at Day 11 onwards for mean tumour size), but the adherent cell population (0–3% T cells, 86–93% B cells) had a low but still significant inhibiting effect ($P<0{\cdot}05$ at Days 11–22 for mean tumour size). In the enhancing experiments, the non-adherent cell population mediated a significantly greater enhancing effect than the adherent cell population (Fig. 6B, $P<0{\cdot}05$ at Day 33 onwards for mean tumour size).

When the 12S lymphocytes were treated with anti-$\theta$ antibody and complement, the results were consistent with those obtained with cells separated on nylon-wool columns. The T-cell-depleted population exerted a smaller effect in both enhancing and inhibiting experiments than 12S lymphocytes treated with complement alone (data not shown).

*Effect of 12S lymphocytes on antigenically distinct tumour cells*

To see whether the effects of lymphocytes were specific or non-specific, these experimental systems were applied to an antigenically distinct transplanted tumour. Neither enhancement nor inhibition of the growth of methylcholanthrene-induced BALB/c fibrosarcoma (MCA-15B) was observed with 12S lymphocytes (Fig. 7).

FIG. 7.—Effect of spleen cells on the growth of MCA-15B cells. 10 mice in each group received $2\times10^6$ lymphocytes per mouse 1 week before (A) or $8\times10^7$ lymphocytes per mouse 1 week after (B) MCA-15B cell inoculation. □——□, 12S lymphocytes; △— · —△, NS lymphocytes; ○ – – – ○, control (medium only). Arrows indicate the time of lymphocyte inoculation. In parentheses: No. mice with tumours/No. inoculated with M-MuSV.

*Infectious centre assay of 12S lymphocytes*

The possibility of virus infection in 12S lymphocytes was examined by the infectious-centre assay. As shown in Table I, low but significant amounts of leukaemia virus (20–40 plaque-forming units (PFU)/$10^6$ cells) and sarcoma virus infections (10–40 FFU/$10^6$ cells) were observed with 12S lymphocytes. However, the T-cell-rich population separated on nylon wool (T cells probably being the main effector cell in inhibiting tumour growth) showed a significantly lower amount of infection (leukaemia virus, 10–20 PFU/$10^6$ cells; sarcoma virus, 5–20 FFU/$10^6$ cells) than the B-cell-rich population (leukaemia virus, 40–50 PFU/$1\times10^6$ cells; and sarcoma virus, 10–60 FFU/$1\times10^6$ cells) ($P<0{\cdot}01$–$0{\cdot}05$ for Expts 2 and 3).

*Effect of virus and lymphoma-cell inoculation on tumour growth*

The effects of inoculation of virus or of antigenically related lymphoma cells (LSTRA) on M-MuSV-induced tumour growth were studied, to see whether the effects of 12S lymphocytes were caused by virus infection of these lymphocytes. The inoculation of the appropriate concentration of M-MuSV ($10^4$ FFU/mouse) inhibited tumour growth, but no enhancing effect was observed. When LSTRA (M-MuLV-induced BALB/c lymphoma) cells were fixed in 0·1% formalin for 1 week (Kudo *et al.*, 1974), washed ×4 in phosphate-buffered saline (PBS) and inoculated

TABLE I.—*Virus Release from LSTRA Cells and from Spleen Cells of Normal and Tumour-bearing Mice: Results of Infectious-Centre Assay*

| Indicator cells | Expt. | No. of focus or plaque formation/$10^6$ cells ± s.d. — Normal spleen | 12S spleen* | Nonadherent 12S† | Adherent 12S‡ | LSTRA (×$10^4$) | $P$ (Nonad <Ad) |
|---|---|---|---|---|---|---|---|
| | 1 | | 41·5±6·4 | | | | |
| D-56 | 2 | 0·6±0·3§ | 38·1±5·5 | 16·8±3·1 | 50·1±4·1 | 1·7±0·2 | <0·001 |
| | 3 | | 24·7±1·5 | 7·3±2·1 | 42·7±2·1 | 2·9±0·9 | <0·001 |
| | 1 | | 13·5±4·9 | | | | |
| BALB/3T3 | 2 | 0·5±0·4‖ | 37·1±4·1 | 18·0±2·3 | 62·5±7·0 | | <0·001 |
| | 3 | | 8·0±1·7 | 3·3±2·5 | 9·7±4·0 | | <0·05 |

* 12S = spleen lymphocytes from tumour-bearing mice inoculated 12 days previously with M-MuSV.
† Consisting essentially of T cells.
‡ Consisting essentially of B cells.
§ Plaque-forming unit ± s.d.
‖ Focus-forming unit ± s.d.

TABLE II.—*Detection of Neutralizing Antibody Activity against M-MuSV with Serum from Mice which Received M-MuSV or Medium only after Inoculation with* 12*S or NS Lymphocytes*

| Serum source | Neutralization titre (V/Vo) Time after lymphocyte inoculation (weeks) | | | | |
|---|---|---|---|---|---|
| | 1 | 2 | 3 | 4 | 5 |
| NS* | 0·97 (0·07)† | 0·72 (<0·01) | 0·86 (0·21) | 0·78 (0·04) | 0·98 (<0·01) |
| NS+M-MuSV‡ | (—) | 0·54 (0·01) | 0·82 (0·16) | 0·69 (0·25) | 0·22§(0·01) |
| 12S‖ | 0·96 (0·13) | 0·56 (0·15) | 0·71 (0·34) | 0·13 (0·10) | 0 (<0·01) |
| 12S+M-MuSV¶ | (—) | 0·60 (0·07) | 0·66 (0·08) | 0·23 (0·09) | 0·19 (0·02) |

* Mice which received normal spleen lymphocytes (NS).
† No. foci in test plate/No. foci in control plate (s.d.).
‡ Mice which received M-MuSV inoculation 1 week after NS inoculation.
§ Underlined figures are significantly different from NS group, $P < 0·01$.
‖ Mice which received spleen lymphocytes from tumour-bearing mice inoculated 12 days previously with M-MuSV (12S).
¶ Group which received M-MuSV inoculation 1 week after 12S inoculation.

i.p. into tumour-bearing or normal mice, neither enhancement nor inhibition was observed (data not shown).

*Neutralization test*

Neutralization tests were performed to detect neutralizing antibody against M-MuSV in serum of mice inoculated with 12S lymphocytes. Ten mice received 12S or NS lymphocytes ($2 \times 10^6$ cells/mouse), and 5 mice in each group were inoculated i.m. with M-MuSV a week after the lymphocyte inoculation. Serum from mice receiving 12S lymphocytes demonstrated earlier neutralizing-antibody activity than that from mice receiving NS lymphocytes (Table II).

### DISCUSSION

The data presented in this paper clearly demonstrate the existence of lymphocyte population(s) in mice with M-MuSV-induced primary tumours, which either enhanced or inhibited tumour growth, depending on the stages of tumour growth at which the lymphocytes were transferred. If lymphocytes collected 12 days after M-MuSV inoculation (designated 12S in our experiments) were inoculated *before* virus inoculation, tumour appearance was delayed and tumour incidence was reduced (inhibiting effect). If the same lymphocytes were inoculated *after* the development of sarcomas, especially early in tumour growth, significant enhancement of tumour growth was observed (enhancing effect). Furthermore, the results indicate that these effects were mediated mainly by the nylon-wool non-adherent, $\theta$-bearing T-cell population.

Why did the same subpopulation of lymphocytes manifest opposite activities when they were inoculated at different stages of tumour growth? Our results may be attributed to a discrepancy between the activity of lymphocytes *in vivo* and *in vitro*. If lymphocytes with tumour-enhancing activity were converted to tumour-inhibiting lymphocytes after their transfer into a recipient animal, inhibition of tumour growth would be observed when the virus was inoculated after the conversion. *In vitro* evidence suggests that spleen cells 12 days after M-MuSV inoculation are in the eclipse stage, as demonstrated by decreased cytotoxic activity against syngeneic M-MuSV- or M-MuLV-induced tumour cells (Hellström and Hellström, 1969; Kirchner *et al.*, 1974). Two weeks after this stage, however, the lymphocytes regain their cytotoxic activity, suggesting a conversion of lymphocyte activity. Another possibility is that the particular expression of lymphocyte activity depends on the condition of the host animals. In contrast to transplanted tumours, the M-MuSV-

induced primary tumours must undergo transformation by virus before the tumours start growing. Thus the inhibiting effect of 12S lymphocytes may be directed only against the sarcoma virus, and the enhancing effect may be directed against the transformed tumour cells.

Another relevant question is whether these enhancing and inhibiting effects are mediated by a single subpopulation of T lymphocytes. Spleen cells from tumour-bearing mice can be fractionated by means of velocity sedimentation into subpopulations capable of inhibiting or enhancing tumour growth (Small and Trainin, 1976). It is conceivable that 2 lymphocyte subpopulations with conflicting activities co-exist in the 12S lymphocytes, and that either one of these activities is expressed under certain conditions of the host animals.

The effect of 12S lymphocytes from M-MuSV-induced sarcoma-bearing mice was compared to that of antigenically distinct tumour cells (MCA-15B). Neither enhancement nor inhibition of tumour was observed with MCA-15B cells. These results, as well as the presence of neutralizing antibody against M-MuSV in the serum of mice inoculated with 12S lymphocytes, suggest some specificity in our findings. Because of the lack of a suitable control for the sarcoma virus, however, we could not study the effect of 12S lymphocytes against other primary tumour systems. Therefore the question whether the effect of 12S lymphocytes is specific to the M-MuSV-induced tumour system or is shared by other primary tumours is still open.

We conclude that the inhibiting effect of 12S lymphocytes was not due to the presence of virus in the 12S lymphocytes because, firstly, virus infection of these lymphocytes was too low to induce such an effect (whereas $10^4$ FFU of virus was required to show significant inhibition, only $10^6$ lymphocytes, which released no more than 40 FFU of virus, manifested significant inhibition). Secondly, the T-cell-rich population, which was probably the main effector population that produced the inhibiting effect, released a lower amount of virus than the B-cell-rich population, according to the infectious-centre assay. Finally, preliminary experiments showed that when 12S lymphocytes (releasing 10–40 FFU of virus/$1 \times 10^6$ cells) were irradiated, they failed to inhibit the appearance of tumours. The earlier appearance of virus-neutralizing antibody in mice inoculated with 12S lymphocytes suggests the participation of antibody formation in the inhibiting effect. Production of cytotoxic T lymphocytes specific to sarcoma cells may also be involved.

In the experiments into the mechanisms of tumour-growth enhancement, the effect of 12S lymphocytes was: (a) observable at the first progressive stage of tumour growth (Fig. 4A); (b) approximately proportional to the numbers of inoculated lymphocytes (Fig. 5B); (c) mediated mainly by the T-cell population (Fig. 6B); and (d) not observed against MCA-15B cells (Fig. 7B). Taking these results into consideration, possible explanations for the mechanisms of enhancement included in our discussion are: (i) non-specific tumour enhancement, (ii) suppressor-cell activity, and (iii) formation of blocking factor.

(i) *Non-specific tumour enhancement*

This has been demonstrated in transplanted tumour systems (Ilfeld *et al.*, 1973; Umiel and Trainin, 1974; Small and Trainin, 1976; Gabizon *et al.*, 1976). Although the failure to enhance antigenically distinct syngeneic tumour cells (MCA-15B) may imply that the mechanism of enhancement in our system is a specific one, it is possible that this failure resulted from the nature of the tumour cells. These cells were growing progressively, and hence might have concealed an enhancing effect. Actually, in some experiments with fast-growing M-MuSV-induced tumours in young mice, we failed to show an enhancing effect on tumour growth. It is unlikely, however, that a cell-free factor released by 12S lymphocytes enhanced tumour growth, because culture medium, collected from 12S lymphocytes and highly concentrated, showed no enhancing effect (unpublished observation).

Although the existence of a lymphocyte population which inhibits tumour growth *in vivo* has not yet been reported (Ilfeld *et al.*, 1973; Nordlund and Gershon, 1975), *in vitro* evidence suggests the presence of cytotoxic T cells and cytostatic B cells in the M-MuSV system (Hellström and Hellström, 1969; Herberman *et al.*, 1974; Lamon *et al.*, 1972; Plata *et al.*, 1974; Leclerc *et al.*, 1972; Lamon *et al.*, 1974; Plata *et al.*, 1976) and the regression of tumours induced by regressor M-MuSV has been attributed to the immune response of the host animals (Fefer *et al.*, 1968; Weinert *et al.*, 1974). In our experiments with progressor M-MuSV, the regressive stage and the first plateau stage observed in adult mice, as well as the occasional regression of tumours, may also be ascribed to the results of a host immune response.

(ii) *Suppressor-cell activity*

Specific suppressor-cell activity of lymphocytes from tumour-bearing animals has been reported in transplanted-tumour systems (Fujimoto *et al.*, 1976*a*, *b*). In the M-MuSV system, suppressor-cell activity of tumour-bearing spleen cells has been reported in *in vitro* stimulation of lymphocytes (Kirchner *et al.*, 1974; Gorczynski, 1974; Kirchner *et al.*, 1975). In our system, enhancement of tumour growth could possibly be mediated by suppressor cells. *Afferent suppression* of immune response (inhibiting the production of cytotoxic or antibody-forming lymphocytes) does not seem to explain the enhancing effect of 12S lymphocytes, because these cells, when inoculated before virus injection, demonstrated an inhibiting effect. However, it is possible that 12S lymphocytes do not suppress antibody formation against the sarcoma virus, but, instead, suppress the production of cytotoxic lymphocytes. Another possibility is that if 2 lymphocyte subpopulations with conflicting activities co-exist in the 12S lymphocytes (cytotoxic killer cells and suppressor cells) suppressor cells may lose their activity in the absence of antigen stimulation (Fujimoto *et al.*, 1976*b*; Basten *et al.*, 1975). Thus, when the 12S lymphocytes are inoculated before the virus injection, the cytotoxic lymphocytes may dominate the suppressor cells in the absence of antigen stimulation, resulting in an inhibiting effect. On the other hand, when the 12S lymphocytes are inoculated after the virus injection, the suppressor cells may dominate the cytotoxic cells in the presence of antigen stimulation, resulting in an enhancing effect.

*Efferent* suppression of immune response (directly inhibiting the effect of immune lymphocytes) may be another possibility. Since 12S lymphocytes release virus, although in small amounts, the 2 agents possibly affecting immune response in this way are virus and virus-producing cells. Their effect was ruled out since the inoculation of M-MuSV ($10^3$–$10^5$ FFU/mouse) or lymphoma cells (LSTRA, $10^5$–$10^7$ cells/mouse) did not enhance tumour growth, even when inoculated after tumour development had begun.

(iii) *Formation of blocking factor*

The induction of blocking factor by the administration of 12S lymphocytes cannot be excluded as a possibility. Enhanced antibody formation induced by the inoculation of 12S lymphocytes may account for their enhancing activity. It is also possible that antibody against 12S lymphocytes, elicited by their inoculation, blocked the specific immune response against tumour cells. Anti-idiotypic antibody formation has been reported in the enhancement of renal allografts in rats (McKearn *et al.*, 1974; Stuart *et al.*, 1976*a*, 1976*b*). Our preliminary experiments indicated that X-irradiated 12S lymphocytes similarly enhanced tumour growth in the M-MuSV system. This finding suggests that the lymphocytes which mediated the enhancing effect were radiation-resistant, or that they served as an antigen to elicit anti-idiotypic antibody in enhancing mechanisms.

Whatever the mechanisms of the enhancing effect of 12S lymphocytes, their elucidation will bring about a better understanding of the reason why tumours grow progressively *in vivo* in spite of the *in vitro* evidence of immune response to the tumour cells.

### REFERENCES

BASTEN, A., MILLER, J. F. A. P. & JOHNSON, P. (1975) T Cell-dependent Suppression of an Antihapten Antibody Response. *Transplant. Rev.*, **26**, 130.

FEFER, A., McCOY, J. L., PERK, K. & GLYNN, J. P. (1968) Immunologic, Virologic, and Pathologic Studies of Regression of Autochthonous Moloney Sarcoma Virus-induced Tumors in Mice. *Cancer Res.*, **28**, 1577.

FUJIMOTO, S., GREENE, M. I. & SEHON, A. H. (1976*a*) Regulation of the Immune Response to Tumor Antigens. I. Immunosuppressor Cells in Tumor-bearing hosts. *J. Immun.*, **116**, 791.

FUJIMOTO, S., GREENE, M. I. & SEHON, A. H. (1976*b*) Regulation of the Immune Response to Tumour Antigens. II. The Nature of Immunosuppressor Cells in Tumor-bearing Hosts. *J. Immun.*, **116**, 800.

GABIZON, A., SMALL, M. & TRAININ, N. (1976) Kinetics of the Response of Spleen Cells from Tumor-bearing Animals in an *in vivo* Tumor Neutralization Assay. *Int. J. Cancer*, **18**, 813.

GLASER, M., KIRCHNER, H. & HERBERMAN, R. B. (1975) Inhibition of *in vitro* Lymphoproliferative Responses to Tumor-associated Antigens by Suppressor Cells from Rats bearing Progressively Growing Gross Leukemia Virus-induced Tumors. *Int J. Cancer*, **16**, 384.

GORCZYNSKI, R. M. (1974) Immunity to Murine Sarcoma Virus Induced Tumors. II. Suppression of T-cell-mediated Immunity by Cells from Progressor Animals. *J. Immun.*, **112**, 1826.

HELLSTRÖM, I. & HELLSTRÖM, K. E. (1969) Studies on Cellular Immunity and its Serum-mediated Inhibition in Moloney-virus-induced Mouse Sarcomas. *Int. J. Cancer*, **4**, 587.

HELLSTRÖM, I. & HELLSTRÖM, K. E. (1970) Colony Inhibition Studies on Blocking and Non-blocking Serum Effects on Cellular Immunity to Moloney Sarcomas. *Int. J. Cancer*, **5**, 195.

HERBERMAN, R. B., AOKI, T., NUNN, M., LAVRIN, D. H., SOARES, N., GAZDAR, A., HOLDEN, H. & CHANG, K. S. S. (1974) Specificity of $^{51}$Cr-release Cytotoxicity of Lymphocytes Immune to Murine Sarcoma Virus. *J. natn. Cancer Inst.*, **53**, 1103.

ILFELD, D., CARNAUD, C., COHEN, I. R. & TRAININ, N. (1973) *In vitro* Cytotoxicity and *in vivo* Tumor Enhancement Induced by Mouse Spleen Cells Autosensitized *in vitro*. *Int. J. Cancer*, **12**, 213.

JULIUS, M. H., SIMPSON, E. & HERZENBERG, L. A. (1973) A Rapid Method for the Isolation of Functional Thymus-derived Murine Lymphocytes. *Eur. J. Immun.*, **3**, 645.

JURIN, M. & SUIT, H. D. (1974) *In vitro* Activity of Lymphocytes and Serum of C3Hf/Bu Mice during the Growth of Methylcholanthrene-induced Tumor and its Regression Following Local Irradiation. *Cancer Res.*, **34**, 672.

KAWASHIMA, K., IKEDA, H., HARTLEY, J. W., STOCKERT, E., ROWE, W. P. & OLD, L. J. (1976) Changes in Expression of Murine Leukemia Virus Antigens and Production of Xenotropic Virus in the Preleukemic Period in AKR Mice. *Proc. natn. Acad. Sci. U.S.A.*, **73**, 4680.

KILBURN, D. G., FAIRHURST, M., LEVY, J. G. & WHITNEY, R. B. (1976) Synergism between Immune Complexes and Serum from Tumor-bearing Mice in the Suppression of Mitogen Responses. *J. Immun.*, **117**, 1612.

KIRCHNER, H., CHUSED, T. M., HERBERMAN, R. B., HOLDEN, H. T. & LAVRIN, D. H. (1974) Evidence of Suppressor Cell Activity in Spleens of Mice Bearing Primary Tumors Induced by Moloney Sarcoma Virus. *J. exp. Med.*, **139**, 1473.

KIRCHNER, H., MUCHMORE, A. V., CHUSED, T. M., HOLDEN, H. T. & HERBERMAN, R. B. (1975) Inhibition of Proliferation of Lymphoma Cells and T Lymphocytes by Suppressor Cells from Spleens of Tumor-bearing Mice. *J. Immun.*, **114**, 206.

KUDO, T., AOKI, T. & MORRISON, J. L. (1974) Stabilization of antigens on surface of malignant cells by formalin treatment. *J. natn. Cancer Inst.*, **52**, 1553.

LAMON, E. W., ANDERSSON, B., WIGZELL, H., FENYÖ, E. M. & KLEIN, E. (1974) The Immune Response to Primary Moloney Sarcoma Virus Tumors in BALB/c Mice: Cellular and Humoral Activity of Long-term Regressors. *Int. J. Cancer*, **13**, 91.

LAMON, E., SKURZAK, H. M. & KLEIN, E. (1972) The Lymphocyte response to a Primary Viral Neoplasm (MSV) through its Entire Course in BALB/c Mice. *Int. J. Cancer*, **10**, 581.

LECLERC, J. C., GORMARD, E. & LEVY, J. P. (1972) Cell Mediated Reaction against Tumors Induced by Oncorna Virus. I. Kinetics and Specificity of the Immune Response in Murine Sarcoma Virus (MSV)-induced Tumors and Transplanted Lymphomas. *Int. J. Cancer*, **10**, 589.

McKEARN, T. J., STUART, F. P. & FITCH, F. W. (1974) Anti-idiotypic Antibody in Rat Transplantation Immunity. I. Production of Anti-idiotypic Antibody in Animals Repeatedly Immunized with Alloantigens. *J. Immun.*, **113**, 1876.

McMASTER, R., BUHLER, K., WHITNEY, R. & LEVY, J. G. (1977) Immuno-suppression of T Lymphocyte Function by Fractionated Serum from Tumor-bearing Mice. *J. Immun.*, **118**, 218.

NORDLUND, J. J. & GERSHON, R. K. (1975) Splenic Regulation of the Clinical Appearance of Small Tumors. *J. Immun.*, **114**, 1486.

PLATA, F., GORMARD, E., LECLERC, J. C. & LEVY, J. P. (1974) Comparative *in vitro* Studies on Effector Cell Diversity in the Cellular Immune Response to Murine Sarcoma Virus (MSV)-induced Tumors in Mice. *J. Immun.*, **112**, 1477.

PLATA, F., MACDONALD, H. M. & ENGERS, H. D. (1976) Characterization of Effector Lymphocytes Associated with Immunity to Murine Sarcoma Virus (MSV)-induced Tumors. *J. Immun.*, **117**, 52.

SJÖGREN, H. O. & HELLSTRÖM, K. E. (1971) Suggestive Evidence that the "Blocking Antibodies"

of Tumour-bearing Individuals may be Antigen–Antibody Complex. *Proc. natn. Acad. Sci. U.S.A.*, **68**, 1372.

SMALL, M. & TRAININ, N. (1976) Separation of Populations of Sensitized Lymphoid Cells into Fractions Inhibiting and Fractions Enhancing Syngeneic Tumor Growth *in vivo*. *J. Immun.*, **117**, 292.

STUART, F. P., MCKEARN, T. J. & FITCH, F. W. (1976*a*) Immunological Enhancement of Renal Allografts by Antireceptor Antibody. *Surgery*, **80**, 130.

STUART, F. P., SCOLLARD, D. M., MCKEARN, T. J. & FITCH, F. W. (1976*b*) Cellular and Humoral Immunity after Allogeneic Renal Transplantation in the Rat. *Transplantation*, **22**, 455.

UMIEL, T. & TRAININ, N. (1974) Immunological Enhancement of Tumor Growth by Syngeneic Thymus-derived Lymphocytes. *Transplantation*, **18**, 244.

WEINERT, C. R., MCMASTER, J. H. & FERGUSON, R. J. (1974) Immune Response to Sarcomas: A Review. *Clin. Orthopaed. Related Res.*, **102**, 207.